# The effects of kisspeptin-10 on serum metabolism and myocardium in rats

**DOI:** 10.1371/journal.pone.0179164

**Published:** 2017-07-10

**Authors:** Ying Zhang, Yuanlong Hou, Xiaoyan Wang, Jihui Ping, Zhiyu Ma, Chuan Suo, Zhihai Lei, Xiang Li, Zheng Zhang, Cuicui Jia, Juan Su

**Affiliations:** 1 College of Veterinary Medicine, Nanjing Agriculture University, Nanjing, China; 2 Ministry of Education Key Laboratory of Systems Biomedicine, Shanghai Center for Systems Biomedicine, and School of Pharmacy, Shanghai Jiao Tong University, Shanghai, China; Max Delbruck Centrum fur Molekulare Medizin Berlin Buch, GERMANY

## Abstract

Kisspeptin is a peptide encoded by the Kiss 1 gene and is also called metastin. Previous studies have generally focused on several functions of this peptide, including metastasis, puberty, vasoconstriction and reproduction. However, few studies have focused on the cardiac functions of kisspeptin. In the present study, cardiac histomorphology was observed via TEM (transmission electron microscope) and HE and Masson staining to observe instinctive changes. Serum metabolites levels were also measured and analyzed using GC/TOF-MS after injection with kisspeptin-10. A gene chip was employed to screen the potential genes and pathways in the myocardium at the transcriptional leve, while RT-PCR and Western Blot were conducted to verify the relevant mRNA and protein expression, respectively. Histopathological findings demonstrated that there were many irregular wavy contractions through HE staining and increased fibrosis around the heart cells through Masson staining after treatment with kisspeptin-10. Additionally, the main changes in ultrastructure, including changes in mitochondrial and broken mitochondrial cristae, could be observed with TEM after treatment with kisspeptin-10. The PCA scores plot of the serum metabolites was in the apparent partition after injection of kisspeptin-10. Twenty-six obviously changed metabolites were detected and classified as amino acids, carbohydrate metabolites, organic acids and other metabolites. Furthermore, gene chip analysis showed 1112 differentially expressed genes after treatment with kisspeptin-10, including 330 up-regulated genes and 782 down-regulated genes. These genes were enriched in several signaling pathways related to heart diseases. The RT-PCR result for ITGB8, ITGA4, ITGB7, MYL7, HIF1-α and BNP corresponded with the gene chip assay. Moreover, the upregulated genes ITGB8, ITGA4 and BNP also displayed consistent protein levels in Western Blot results. In summary, these findings suggest that kisspeptin-10 could alter the morphology and structure of myocardial cells, serum metabolite levels, and expression of genes and proteins in heart tissues. Our work determined the profound effects of kisspeptin-10 on the heart, which could further lead to the development of therapeutics related to kisspeptin-10, including antagonists and analogs.

## Introduction

Kisspeptin is a polypeptide composed of 145 amino acids and encoded by the KISS1 gene in human, or Kiss1 gene in animals[1]. It has been revealed to be cleaved by proteases into shorter peptides known as KP-54, KP-14, KP-13 and KP-10[2]. These smaller fragments retain similar biological activities, which are most likely related to the polypeptide C-terminal region [3]. These peptides pair with the G-protein coupled receptor GPR54[4]. Previous studies have generally focused on a wide range of functions, including roles in suppressing tumor metastasis[5], controlling reproduction with respect to initiating puberty onset[6] and as an important component for controlling the hypothalamo-pituitary-gonadal (H-P-G) axis by regulating gonadotropin-releasing hormone (GnRH)[7].

It was recently reported that kisspeptins, including KP-10, KP-13, and KP-54, were identified in the human coronary artery and umbilical vein and had a potent endothelium-dependent vasoconstriction function; its responses were comparable to that of the vasoactive peptide Ang-II[3]. It was also demonstrated that injection of kisspeptin-10 could slow down microvascular cutaneous blood flow in mice[8]. Moreover, kisspeptin receptors were found in atrial and ventricular tissues of humans and then were detected in rat and mouse, with the effect of eliciting intropic activity on paced atrial strips[9]. Its role in contraction has been proven to be very potent, even comparable to endothelin and apelin[9, 10]. To our knowledge, circulating kisspeptin concentrations in healthy individuals are very low. Patients experience hypogonadotropic hypogonadism and delayed puberty, but do not develop any cardiovascular phenotypes[6, 9]. However, circulating kisspeptin concentrations were significantly higher (10,000 times) in pregnancy compared with non-pregnant patients[11]. Thus, some researchers speculate that kisspeptin might contribute to the adaptive increase in cardiac output[12]. Moreover, there is evidence that that kisspeptin is involved in the pathogenesis of pre-eclampsia. This disease displays hypertensive symptoms that are induced by artery constriction. During the development of pre-eclampsia, gestational hypertension heart disease is characterized by myocardial injury, which is a serious complication. These previous studies speculate that kisspeptin may play more roles than just affecting myocardial contraction. To obtain significant evidence, it is highly important to adopt more sensitive and specific approaches for determining the mechanism of variation induced by kisspeptin-10 treatment.

Metabolomics is considered a quantitative measurement of the dynamic multiparametric metabolic responses of living systems to pathophysiological stimuli or genetic modification[13]. It is analyzed with endogenous global metabolites obtained from both tissues and biological fluids[14, 15]. In previous studies, metabonomics has been applied to study many types of heart diseases. Yue-tao Liu developed an UPLC/QTOF-MS-based metabonomics approach to identify the metabolic pathways involved in the protective actions of XKS in myocardial infarction[16]. Fang Li applied NMR-based metabonomics to evaluate the effects of AMB on acute myocardial ischemia[17]. Yu Chen used GC/MS and UPLC/MS/MS to explore the possible pharmacological mechanism of SMI’s action and the pathogenesis of DOX-CM[18]. Additionally, myocardial dysfunctions, including left ventricular hypertrophy, myocardial ischemia and heart failure, have been considered to be frequently associated with changes in cardiomyocyte metabolism[19–21].

In the present work, we investigated the changes in cardiac metabolites in rats treated with kisspeptin-10 using a metabonomics approarch based on GC/TOF-MS to the rats. The identification of metabolic pathways and biomarkers may contribute to understanding the mechanism by which kisspeptin-10 treatment alters cardiac functions. Mitochondria in which energy metabolism occurs was observed through transmission electron microscope (TEM), and the perturbations in energy metabolism could be inferred from changes in mitochondrial structure. We next developed an mRNA microarray analysis to screen the transcriptional levels of various genes and biomarkers and subsequently confirmed the protein expression levels of key genes. Thus, in this study, we aimed to determine the effects of kisspeptin-10 on cardiac functions in rats from multiple perspectives.

## Materials and methods

### Animals and sample collection

All of the animal procedures were approved by the Institutional Animal Care and Use Committee of Nanjing Agricultural University (project number: 2009ZX08009-143B). Sampling procedures were performed in strict accordance with the Guidelines on Ethical Treatment of Experimental Animals of China. Twenty eight-week-old male Sprague-Dawley rats weighing 200 ± 20 g were obtained from Shanghai Laboratory Animal Center (SLAC, Shanghai, China). They were maintained at constant temperature (23 ± 2°C) and humidity (45 ± 15%), with controlled lighting (light 12 h—dark 12 h), and were fed at a standard diet, with free access to tap water. They were adapted to the environment for two weeks to overcome the stress of transportation before the animal experiments began. Then, all of the rats were divided into two groups randomly. Each rat in kisspeptin-10 group received subcutaneous injection with 200 μL kisspeptin-10 (40 nmol/200μL) every day, each rat of the control group received subcutaneous injection with 200 μL saline every day, both of the two groups were treated for 7 days continuously. Rats were anesthetized with urethane (1.5 g/kg, i.p.). Under general anesthesia, blood samples were collected and rats were euthanized. All efforts were made to minimize the discomfort and stress of animals. Then all the samples were analyzed. Kisspeptin-10 was purchased from TOCRIS Bioscience Company (Bristol, UK) and dissolved in physiological saline. Seven days later, the 20 rats were decapitated after exposure to anesthesia. Cardiac tissue and serum samples were collected and stored at -80°C until being used for total RNA and protein extraction as well as metabonomics analysis. The rest of the heart tissue was fixed with 4% paraformaldehyde and 2.5% glutaraldehyde for 24 h at 4°C for further morphological research.

### Morphological observation

Transmission electron microscope (TEM): The heart tissues fixed with 2.5% glutaraldehyde were chopped into 0.1 cm^2^ × 1 cm pieces. Then, the tissues were rinsed, dehydrated, embedded, sliced, stained, and subsequently investigated under a transmission electron microscope (TEM). Under a 20,000× magnification by TEM, the degree of mitochondrial damage in the two groups was estimated according to the mitochondrial damage classification method named as Flameng. About 20 mitochondrial from each of 5 randomly selected fields (100 mitochondria per rat) were analyzed. The mitochondrial damage types were assessed using a scale from 0 to 4 with 0 indicating a normal structure, 1, normal with slight swelling, 2, mitochondrial swelling, 3,serious swelling and cristae disorder, 4, mitochondria membrane breach and vacuolization. On the basis of the above scale, the degree of mitochondrial damage of rats between the two groups was scored and the total scores of 100 mitochondria per rat were summed. Finally, this value was divided by 100, and the ratio accounted for the degree of mitochondrial damage. The higher the ratio is, the more severe the injure is[22].

Hematoxylin and eosin (HE) staining: The heart tissues fixed with 4% paraformaldehyde were chopped into 0.5 cm × 0.5 cm × 1 cm pieces. Then, samples were dehydrated until transparent, immersed in wax, and embedded in paraffin. The paraffin-embedded tissues were cut into 5 μm slices using a Rotary Microtome YD-1508R (Jinhua YIDI Medical Appliance Co., Ltd, China). Then, the slices were routinely stained with hematoxylin and eosin (HE). Next, the stained sections were observed under an OLYMPUS BX43 microscope (Olympus Co., Japan) to compare the changes in the myocardium microstructure between the control and the kisspeptin-10-treated groups.

Masson’s trichrome staining: The Masson assay was used to detect fibrosis levels in normal and kisspeptin-10-treated cardiac tissue samples. The prepared paraffin sections were stained with Masson’s trichrome and then observed through an OLYMPUS BX43 microscope (Olympus Co., Japan).

### GC/TOF-MS sample preparation and analysis

Serum samples were first derivatized according to published methods with few modifications and analyzed with GC/TOF-MS[23, 24]. Briefly, the two internal standards (10 μL of 0.3 mg/mL L-2- in water and 10 μL of 1 mg/mL heptadecanoic in acid methanol) were spiked into 200-μL serum samples from each rat in sequence, followed by a 30-s vortex and 10-min storage at -20°C. After the extraction procedure, the mixtures were centrifuged at 13,200 rpm for a period of 10 min, of which only 200 μL of supernatant was transferred to each GC sampling vial for vacuum drying at 20°C. Next, the residues were derivatized with a two-step procedure where they were added to 50 μL 15 mg/mL methoxyamine in pyridine for 90 min at 30°C and then to 80 μL BSTFA (containing 1% TMCS) for 60 min at 70°C. Afterwards, 1 μL of the derivatized solution from the last step were injected into an Agilent 6890N gas chromatograph that was coupled with a Pegasus HT time-of-flight mass spectrometer (GC/TOFMS, Leco Corp., Joseph, MI) in splitless mode at 260°C. The metabolite separation flow used helium as the carrier gas and was achieved in a DB-5 ms capillary column (Agilent J&W Scientific, Folsom, CA). The constant flow speed was set at 1.0 mL/min. Meanwhile, we began the GC temperature program with a solvent delay for 5 min and the following steps: (1) in order: 80°C for 2 min, (2) 10°C/min ramp to 180°C, (3) 6°C/min ramp to 230°C, (4) 40°C/min ramp to 295°C, and (5) a final maintenance step for 8 min. The TOFMS aquisition rate was set at 20 spectra/s in full scan mode with 30 to 600 m/z electron ionization.

Statistical analysis: Chroma TOF software v4.22 (Leco) were used for the multivariate analysis after the data from the GC/TOF-MS mass spectrogram and chromatogram were pretreated. All of the procedures were conducted according to previous publications[25]. Then, the final dataset was obtained, from which artificial peaks with noise peaks, column bleed, and BSTFA (N, O-bis trifluoroacetamide) were removed. Metabolite identification was performed with SIMCA-p (13.0) software using PLS-DA and PCA models. Data reduction approaches such as unsupervised approach PCA or supervised approach PLS-DA, can help to reduce complex data to a manageable form. The PCA was first used in all samples to see the general separation and to find the outliers, then followed by the PLS-DA. In this study, the PCA demonstrated a distinct separation across the control group and kissppetin-10 group, suggesting the existence of different metabolic characteristics induced by the kisspeptin-10 treatment. PLS-DA picks out discriminating ions that are contributing to the classification of samples and remove noncorrelated variations contained within spectra. The VIP (variable importance) values were considered differential variables when they were greater than 1.0. Fold change values were adopted to further select and validate the differentiating variables.

### mRNA expression profile microarray

We harvested the rat heart tissues from groups N and K were harvested and pooled them by groups for RNA extraction. The TAKARA mRNA Isolation Kit (TAKARA, Japan) was used according to the manufacturer’s instructions for this procedure. Total RNA for further analysis was purified before using a RIN number check to guarantee the RNA integrity with an Agilent Bioanalyzer 2100 (Agilent technologies, Santa Clara, CA, US). Then, the qualified total RNA was prepared with an RNeasy mini kit (QIANGEN, GmBH, Germany) and an RNase-Free DNase Set (QIANGEN, GmBH, Germany). The gene expression chip used in this research was purchased from Agilent technologies (US) by the SBC (Shanghai Biotechnology Corporation, China), and the scanning of biochips was performed with an Agilent biochip scanner (Agilent Microarray Scanner, Agilent, USA). Data reading and standardizing analyses were conducted with the Feature Extraction software 10.7 and normalized with the Gene Spring Software 11.0 (Agilent, USA). Finally, the gene ontology (GO) database and the Kyoto encyclopedia of genes and genomes (KEGG) were used to classify the biological processes and determine the signal pathways enriched by the differentially expressed genes enriched in the groups K and N.

The bioinformatics classification was confirmed with bioinformatic analysis tools, the gene ontology (GO) database and KEGG (Kyoto Encyclopedia of Genes and Genomes)[26].

### Quantitative real-time PCR

Total RNA from the two groups was extracted with the Tissue RNA kit (Biomiga, USA). Then, cDNA was obtained through reverse transcription using the Reverse Transcriptase kit according to the manufacturer’s protocol (Reverse Transcriptase for qPCR, Vazyme, China). The gene primers were designed according to the gene sequences from GeneBank with Primer 3.0 and the NCBI-BLAST primer designing tools. The forward (F) and reverse (R) primers for the genes are listed in Table 1. All of the primers were synthesized by Life Technologies Corporation (Shanghai, China).

**Table 1:** Primers used for real-time PCR.

Taget genes	Genebank accession NO.	Primer sequence (5’-3’)	Product lengh (bp)
ITGA4	NM_001107737	F: GCATCGTGTCAAGCTGGAAT	199
		R: TTCGGAAATGACCAGCTCCT	
ITGB7	NM_013171	F: AAGAGAAGGGAGCAGACCAC	151
		R: TGCTGCCTCTCCTTCTCAAA	
ITGB8	NM_001108726	F: GACTCAAAGGACAGTGTGCG	153
		R: AGATACAAACACCTCGGCCA	
MYL7	NM_001106017	F: CCTTCAGCTGCATTGACCAG	182
		R: CCCATTGAGTTTCTCCCCGA	
BNP	NM_031545	F: CCAGAACAATCCACGATGCA	192
		R: GCAGCTTGAACTATGTGCCA	
HIF-1α	NM_024359	F: ACAGAAATGGCCCAGTGAGA	201
		R: TAGGCGGTTTCTTGTAGCCA	
GAPDH	NM_017008	F: ATGGTGAAGGTCGGTGTGAA	175
		R: TGACTGTGCCGTTGAACTTG	

Quantitative real-time PCR was performed using the SYBR^®^ Green Master Mix kit (Vazyme, China) on an Applied Biosystems QuantsStudio-5 Real-Time PCR system (Thermo Fisher, USA). The reaction volume, procedure, and conditions were based on the SYBR Green Master Mix instructions. Each sample set included 3 repeats, and the results for relative gene expression were determined using the 2^-ΔΔCt^ method, which were all normalized to GAPDH expression.

### Western blot

Heart tissue protein extraction and determination of protein concentration: 100 mg of heart tissue per rat was placed in a 2.0-mL centrifuge tube, and 1 mL PIPA cracking liquid with 10 μL PMSF inhibitors was added to the tube. Then, the mixture was homogenized on ice and centrifuged at 16,000 g/min for 5 min at 4°C. The liquid layer, which contained the total protein, was saved and transferred to a new tube. The protein concentration was detected with the Total Protein Assay kit (with standard: BCA method, Beyotime Biotechnology, China) according to the manufacturer’s instructions. The total protein solutions were adjusted to the same concentration (10 μg/μL) with 5×SDS sample buffer, which was eventually dissolved to 1×SDS. Finally, the protein samples were boiled for 10 min and saved at -20°C for the following research.

Electrophoresis, Western Blot and Detection: Firstly, 75 μg total protein was added to each well and separated via SDS-PAGE through a stacking (5%) and separation (10%) gel in order. Then, the separated proteins were transferred to a NC (Nitrocellulose) membrane via a Trans-blot SD (VE 386, Tanon, China) at 40 mA for 75 min. Next, the membranes were blocked for 2 h with 5% skim milk powder diluted in TBST (Tris Buffered Saline with Tween-20) at room temperature, followed by incubation with primary antibodies for 12 h and then secondary antibodies for 2 h. The following primary and secondary antibodies at varying dilutions were used in the Western-Blot experiments: (1) GAPDH monoclonal antibody-HRP (Bioworld Technology, Inc.) diluted 1:10000 with TBST; (2) ITGB8 rabbit polyclonal antibody (Santa Cruz, USA) diluted 1:1000 with TBST; (3) ITGA4 rabbit polyclonal antibody (Santa Cruz, USA) diluted 1:500 with TBST; (4) brain natriuretic peptide (BNP) goat polyclonal antibody (Santa Cruz, USA) diluted 1:500 with TBST; and (5) Goat anti-rabbit IgG-HRP and rabbit anti-goat IgG-HRP (TRANS, China) diluted 1:5000 with TBST. Then, the membranes were washed with TBST for 30 min, followed by detection with chemiluminescence and image densitometry analysis with a Tanon-6200 luminescent image analysis system (Tanon-6200, china) with BeyoECL Plus (Beyotime Biotechnology, China).

### Statistical analysis

The statistical significance of all the data, which are presented as the mean ± SEM, was evaluated using the independent samples T test with SPSS 17.0 software (SPSS, IBM^®^, NY, USA). P value less than 0.05 was considered to be a significant difference.

## Results

### Morphological evaluation

#### Transmission electron microscope (TEM)

**Fig 1:**
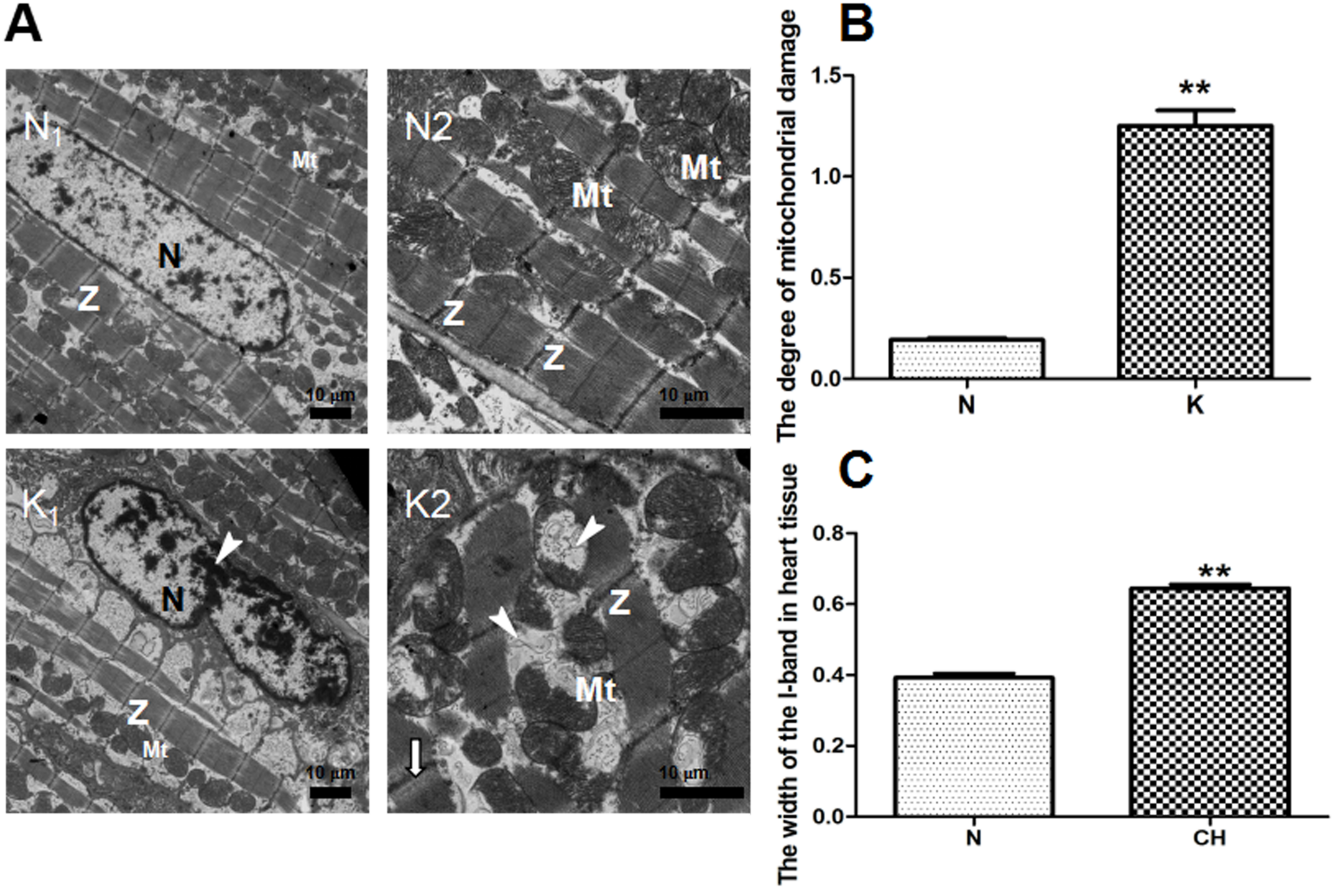
Heart tissue ultrastructure of the N and K groups by transmission electron microscope (TEM) and myocardial cell quantitative analysis of ultrastructural damege effects of kisspeptin-10 on mitochondria from group K and group N. A: (N_1_) (N×1.2 k) The normal cardiomyocyte nuclei (N) and clear myocardial Z-band (Z) from the N group. (N_2_) (N×3.0 k) Normal mitochondria (Mt) from the N group. (K_1_) (K×1.2k) Margination of nuclear chromatin (↓) in the K group. (K_2_) (K×3.0 k) Injured mitochondrial cristae(▼) in the K group. B: The degree of mitochondrial damage. N: 0.19 ± 0.125, K: 1.25 ± 0.108. C: The width of the I-band in heart tissue. N: 0.393 ± 0.01 μm, K: 0.645 ± 0.01 μm, ** *P* < 0.01.

**Fig 2:**
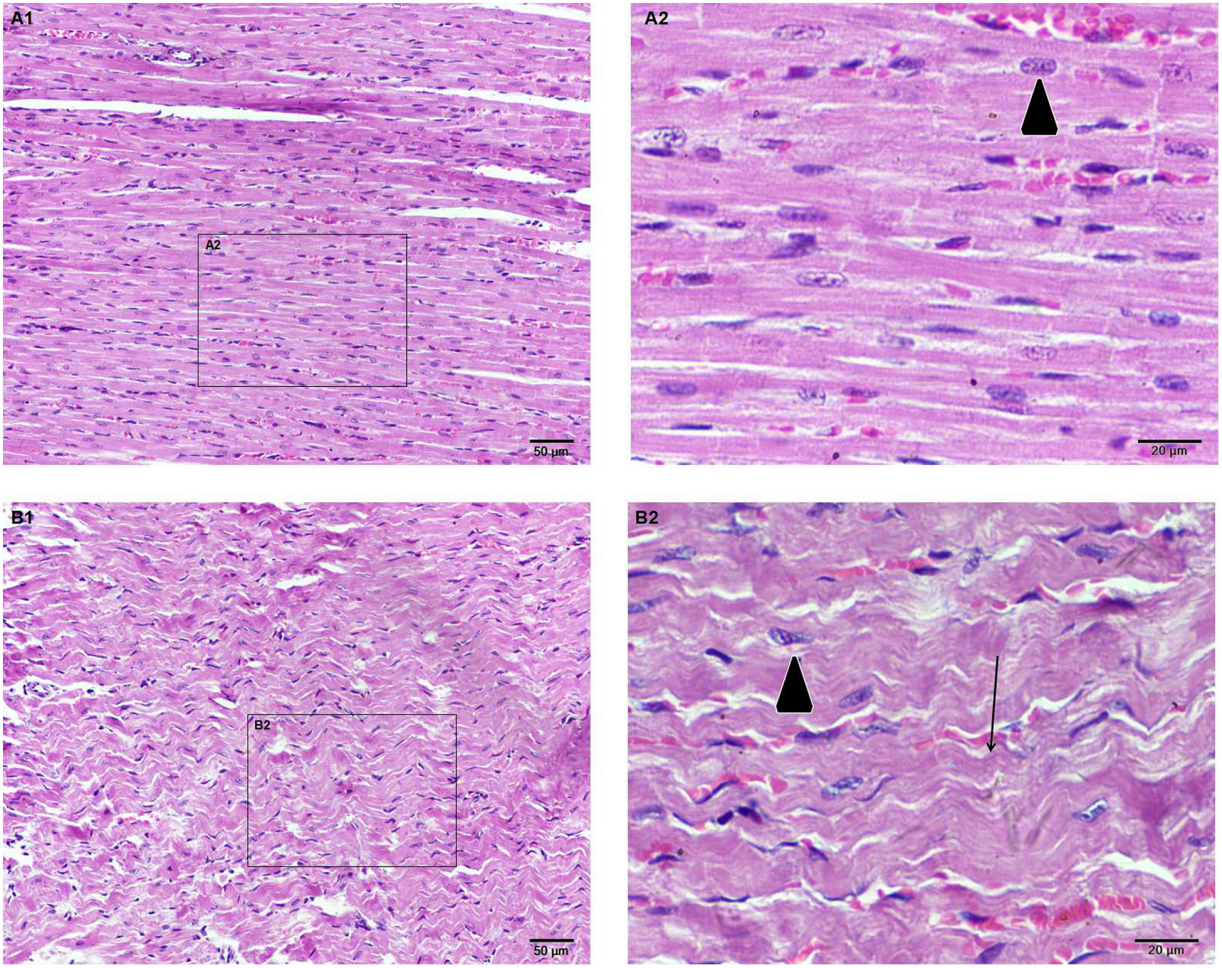
Light microscopy of heart tissues from the two groups stained with hematoxylin-eosin. (A1) (N×200) Myocardial fibers are neatly arranged. (A2) (N×400) Homogenous cytoplasmic and nuclear staining(▼). (B2) (K×400) A marked contraction in myocardial cells (**↓**) and uneven dyeing of myocardial cells(▼).

Kisspeptin-10’s effects on the myocardium are shown in Fig 1(A), 1(B) and 1(C). A: The control group (N) displays intact cardiac muscle cells, clear myocardial fiber textures, neat rows, and the shape of the mitochondria cristae looked normal and complete. Through the Flameng method, the degree of mitochondria damage of heart tissues from group K and group N was valued and demonstrated in Fig 2(B), the higher the scroe is, the more svere the injure is. The scroe in the group K was significantly higher than the control group (*P* < 0.01), which indicated the severe damage of mitochondria in group K induced by kisspeptin-10. In the group K, the I-band became wider compared to the normal cells, which was analyzed quantitatively and demonstrated in Fig 1(C). The width of I-band in group K was widened significantly compared to the control group (*P* < 0.01). We also observed some uncommon characteristics, including disorderly arranged cardiac muscle fibers, margination of nuclear chromatin and fractured and even partly missing mitochondria cristae.

#### Hematoxylin eosin (HE) staining

Kisspeptin-10’s effects on the myocardium microstructure are shown in Fig 2. The myocardial fibers in the control group (N) are neatly arranged (Fig 2-A1). Moreover, the cytoplasm and nuclei in the cardiac muscle cells were homogenously stained in the high magnification image (Fig 2-A2). In the kisspeptin-10 group (K), the morphology of myocardial cells appeared markedly contracted (Fig 2-B1). Additionally, there were slightly shorter sarcomeres and the cytoplasm showed uneven staining at high magnification (Fig 2-B2) compared to the control group (N).

#### Masson staining

**Fig 3:**
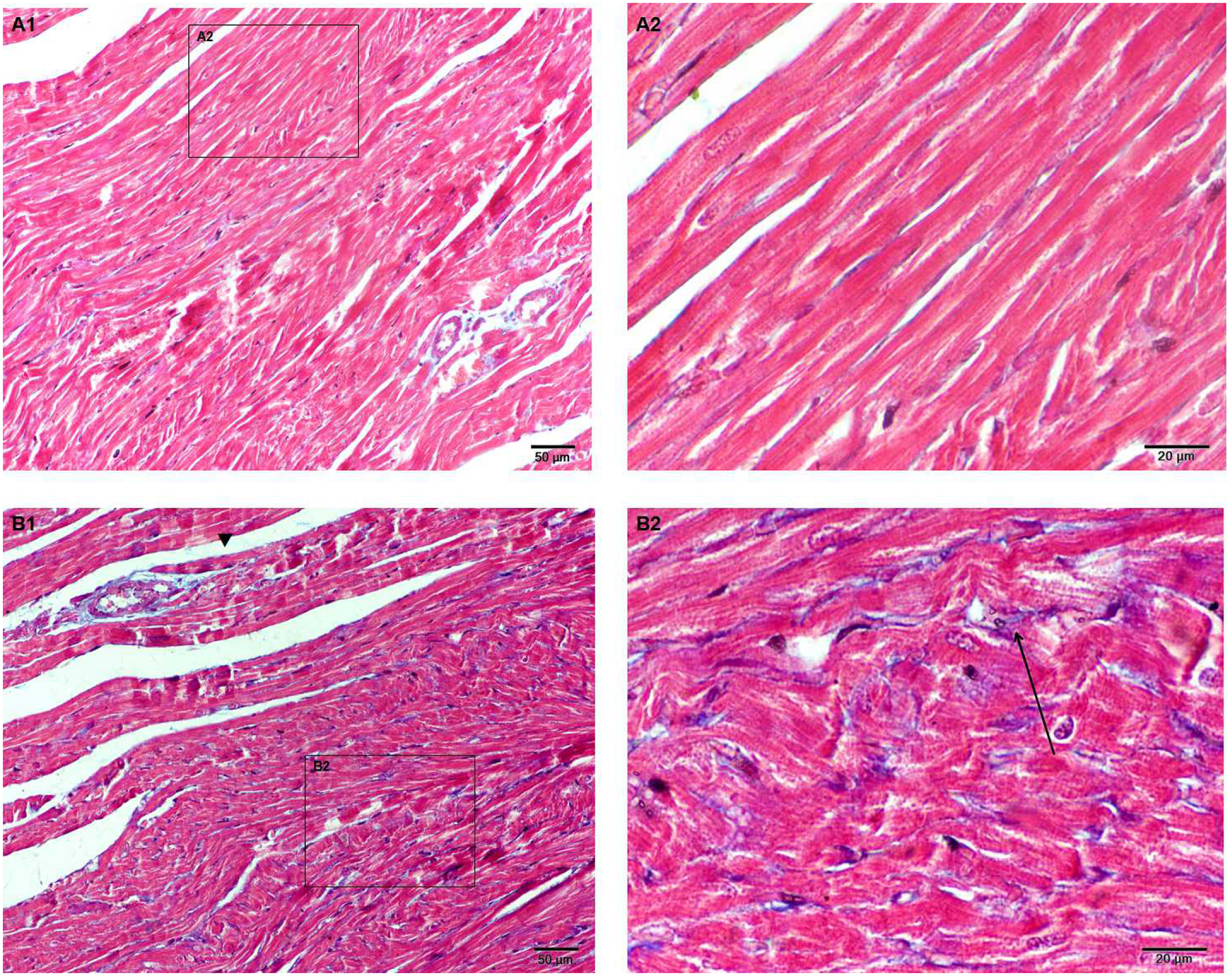
Myocardial fibrosis in heart tissue via Masson staining in the N and K groups. (A1) (N×200) The normal distribution of myocardial fibrosis. (A2) (N×400) Myocardial fibers are neatly arranged. (B1) (K×200) Collagen fibers hyperplastic state in heart tissue from group K. Collagen fibers in a hyperplastic state around blood vessels (▼). (B2) (K×400) A high magnification image of collagen fiber hyperplasia around a myofiber (**↑**).

Masson collagen staining shows the hyperplastic state of heart fibers between the control (N) and kisseptin-10 (K) groups (Fig 3). Compared to the control group (N), there was a degree of fibrosis in heart cells from the kisspeptin-10 group (K).

### The metabolic effects of kisspeptin-10 on rats

**Fig 4:**
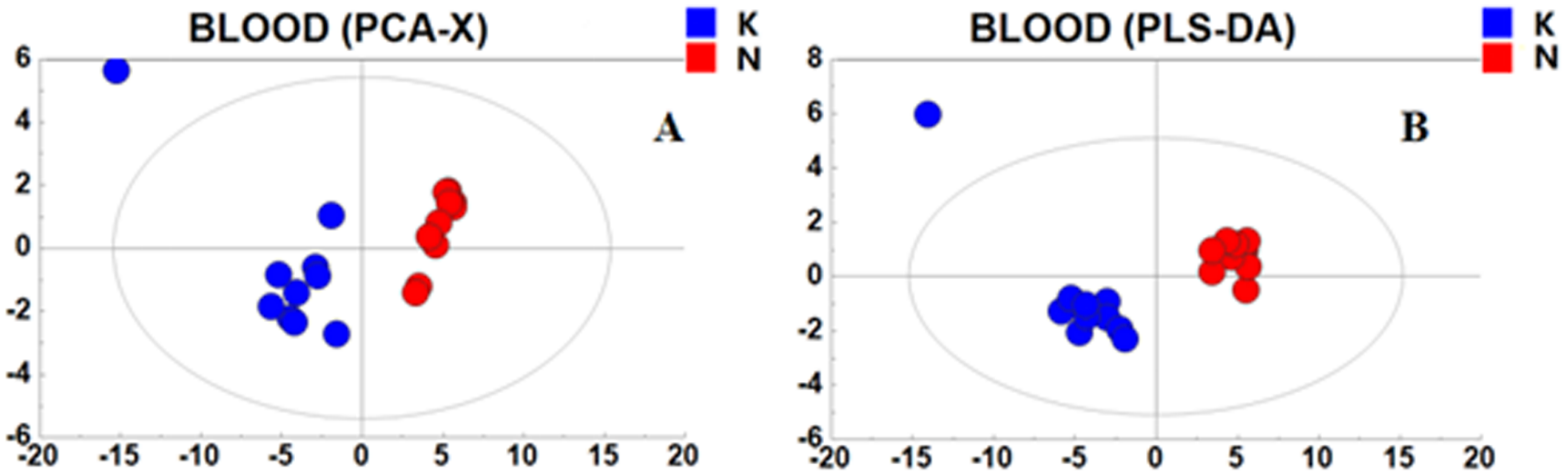
PCA and PLS-DA scores plot for rat serum in the N and K group. (A) PCA scores plot for the N and K groups for rat serum. The red dot is the control group (N), and the blue dot is the kisspeptin-10 group (K). (B) PLS-DA scores plot for the N and K groups for rat serum. The red dot is the control group (N), and the blue dot is the kisspeptin-10 group (K). (A) R2X[1] = 0.673, R2X[2] = 0.0832, Ellipse: Hotelling’s T2 (95%). (B) R2X[1] = 0.671, R2X[2] = 0.0771, Ellipse: Hotelling’s T2 (95%).

The PCA scores plot of the two groups is shown in Fig 4. There is a clear separation between the control (N) and kisspeptin-10 (K) groups in the plot regarding serum samples. This finding suggests that changes in the serum metabolites might be caused by kisspeptin-10 treatment. An additional PLS-DA scores plot was generated for PCA from the GC/TOFMS data. In the PCA and PLS-DA maps, each spot represented a sample and each assembly of samples indicated a particular metabolic pattern of the two groups. (Fig 4).

**Fig 5:**
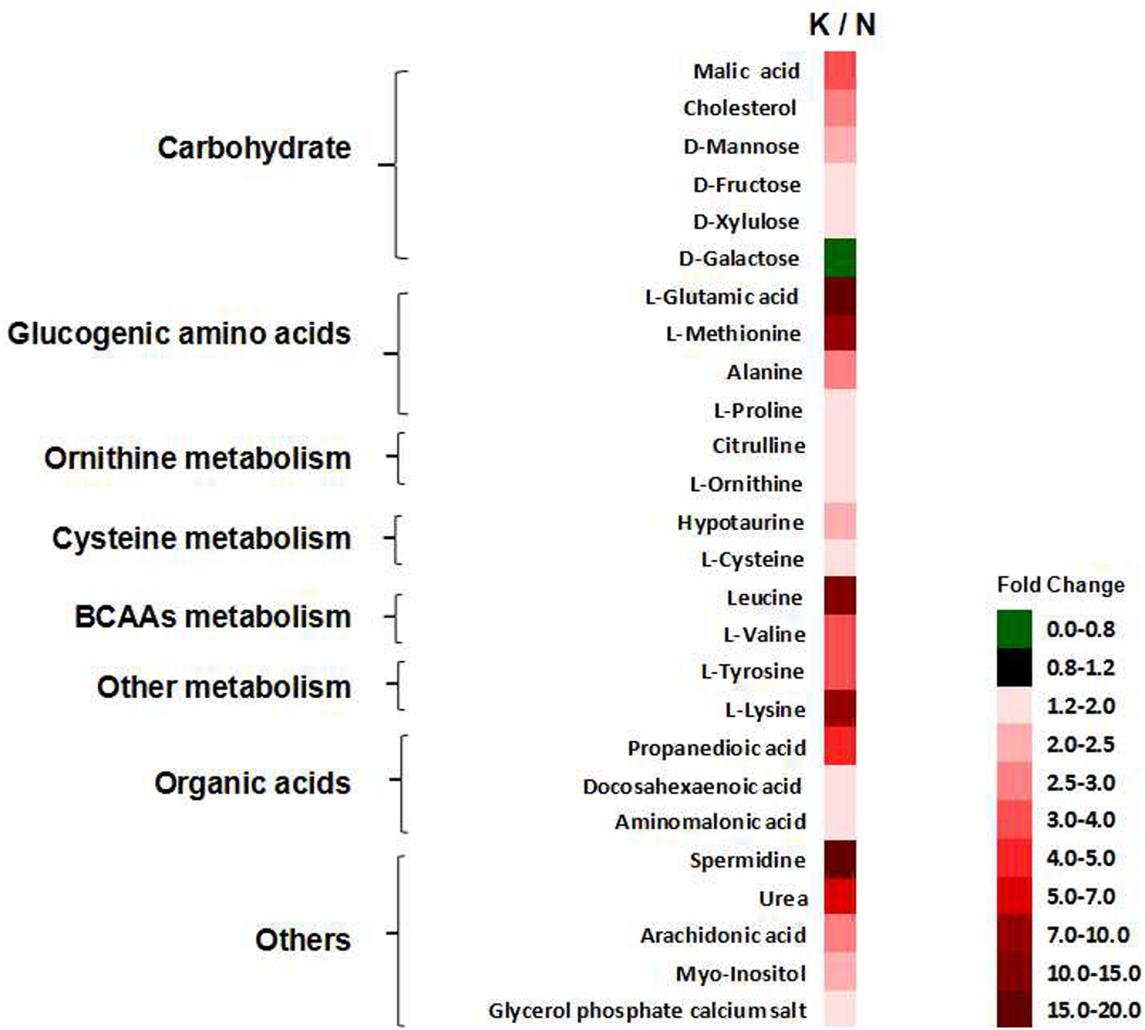
The 26 differentially expressed metabolites in the N and K groups. The fold changes of the arithmetic mean value (*P* < 0.05) of kisspeptin-10-induced changes in serum metabolites. ([N] control group and [K] kisspeptin-10 group).

There were 26 differentially expressed serum metabolites identified in the two groups (Fig 5), and the ANOVA and Kruskal-Wallis testes were performed with a significance threshold at P = 0.05. Fold changes obtained from the ratio of arithmetic mean values for the metabolites in the kisspeptin-10 group (K) were calculated and compared to those in the control group (N).

### The effect of kisspeptin-10 on gene expression

The Agilent 4×44k genome-wide Oligo chip detection for the control (N) and kisspeptin-10 (K) groups was performed to screen for the potential genes associated with heart function. We discovered that 330 genes were upregulated and 782 genes were downregulated by more than 2-fold (*P* < 0.05).

The analysis of and KEGG pathway, The signal pathways that included the differentially expressed genes were determined using the GO function and KEGG pathway analyses (*P* < 0.05). The altered enriched pathways and genes related to heart functions are listed in Table 2.

**Table 2:** Screened cardiomyopathy related pathways and genes according to the result of chip.

Cardiomyopathy related pathways	Changes in genes
Regulation of actin cytoskeleton	Itgb7, Itgam, Itga4, Itgal, Vav1, Itgb2, Pik3r3, Pak1, Cyfip2, Iqgap2, Was, Itgb8, Myl7
HIF-1 signaling pathway	Timp1, Hk3, Cybb, Pik3r3, Cdkn1a, Nox1, Nppa
Focal adhesion	Itgb7, Itga4, Vav1, Spp1, Figf, Pik3r3, Pak1, Tnc, Itgb8, Myl7,
Tight junction	Cldn2, Cldn4, Hcls1, Prkci, Magi2, Myl7,
Hypertrophic cardiomyopathy (HCM)	Itgb7, Cacna1d, Itga4, Cacng2, Itgb8

### Relevant mRNA expression

**Fig 6:**
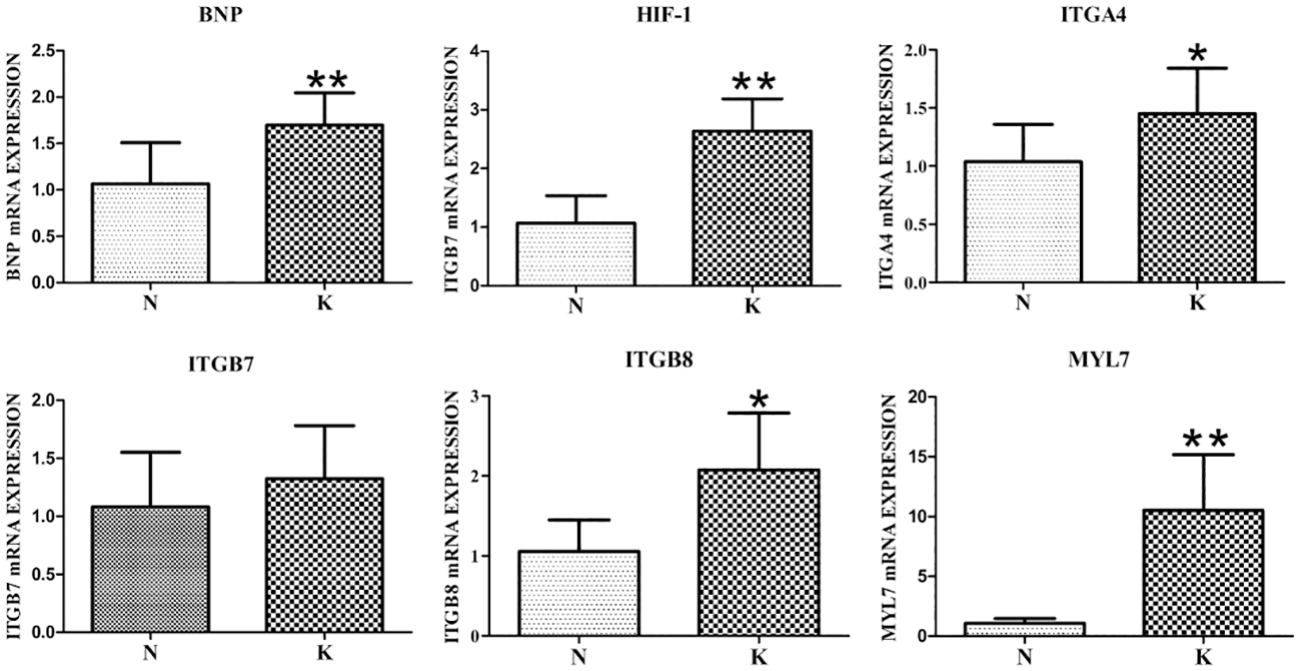
Expression of mRNAs associated with cardiac diseases in the kisspeptin-10 and control groups in rats. (** *P* < 0.01 and * *P* < 0.05. [N] control group and [K] kisspeptin-10-treated group. n = 5 per group).

The differentially expressed genes were primarily screened from the gene chip. Some of those genes, including ITGA4, ITGB8, MYL7, ITGB7, HIF-1α and BNP, are assosiated with heart disease pathways and are recognized as myocardial injury biomarkers. The expression of these genes was detected with an RT-PCR assay and is shown in detail in Fig 6. In heart tissue, integrin subunit beta 8 (ITGB8) and integrin subunit alpha 4 (ITGA4) mRNA expression increased significantly (*P* < 0.05) in the kisspeptin-10 group (K) compared with the control group (N) (Fig 6). Myosin light chain 7 (MYL7), brain natriuretic peptide (BNP) and hypoxia inducible factor 1 alpha subunit (HIF-1α) mRNA expression was very significantly (*P* < 0.01) increased in the kisspeptin-10 group (K) compared with the control group (N) (Fig 6).

### Western blot analysis after kisspeptin-10 treatment

**Fig 7:**
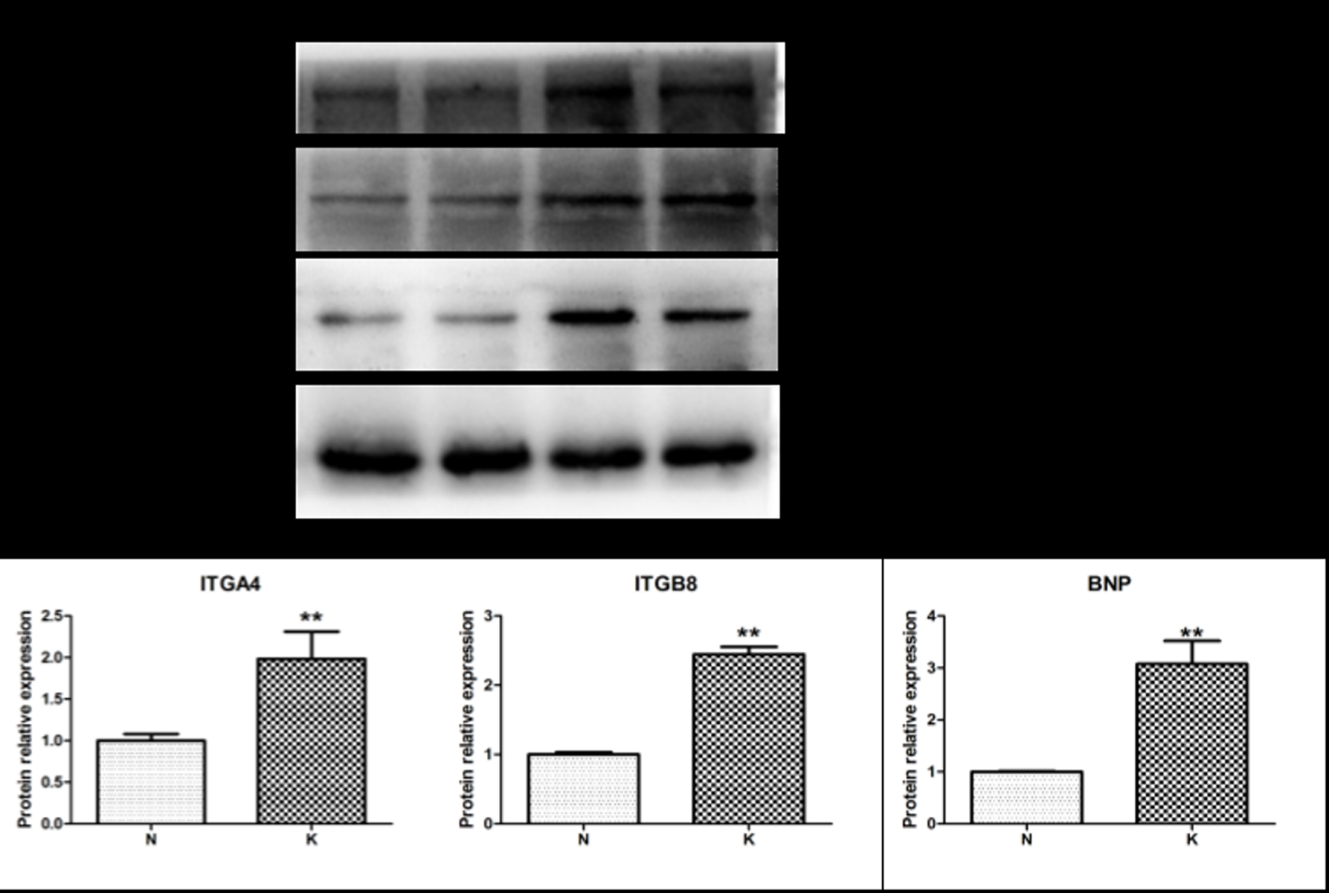
Itga4, Itgb8, and BNP protein expression in heart tissue from the kisspeptin-10 and control groups in rats. (** *P* < 0.01. [N] control group and [K] kisspeptin-10-treated group.)

Western blot was performed to evaluate the effects of kisspeptin-10 treatment on ITGA4 and ITGB8 protein expression in heart tissue from the two groups, as these proteins are involved in heart function pathways. The results showed that ITGB8 and ITGA4 protein expression significantly (*P* < 0.05) increased in the kisspeptin-10 group (K) compared with the control group (N) (Fig 7). Moreover, the protein expression of the heart disease biomarker BNP significantly increased in the kisspeptin-10 group (K) compared with the control group (N) (Fig 7).

## Discussion

Previous studies on kisspeptins have mainly focused on characterizing their responses in puberty, reproduction and tumor metastasis[27]. To date, few studies have examined their cardiovascular effects. Currently, kisspeptins have been shown to be potent vasoconstrictor compounds and to promote contraction in myocardial cells in the cardiovascular system[3, 9]. It has also been reported that kisspeptin-10 can decrease peripheral blood flow and induce plasma extravasation[8]. Another study has shown that kisspeptin and its corresponding receptor are expressed in rat, mouse and human hearts. Additionally, kisspeptins have been shown to be potent positive inotropes in vitro cardiac preparations[9].

A large number of studies have indicated that the kisspeptin receptor coupling to Gq/11 leads to phospholipase C activation and Ca^2+^ release[2, 28], as well as activation of Rho and Rho-associated kinase[29]. However, the specific mechanisms by which these activation are induced by kisspeptin-10 remain to be investigated. In our study, these pathways were detected by gene chip and analyzed with KEGG pathway analysis. The results identified several pathways involved in heart diseases. The two differentially expressed genes ITGA4 and ITGB8 are present in heart disease-related pathways, including Dilated Cardiomyopathy (DCM), Arrhythmogenic Right Ventricular Cardiomyopathy (ARVC), and hypertrophic cardiomyopathy (HCM). The expression of these two genes was upregulated at the transcriptional and protein levels after kisspeptin-10 treatment in our study.

ITGA4 and ITGB8 belong to the integrin family, which includes important cell surface molecule receptors that mediate cell-cell and cell-extracellular matrix interactions[30]. Integrins are distributed across the intercalated discs and Z-bands in cardiac myocytes[31]. Previous research has shown that increased heart integrin expression during pressure overload indicates a potential role in hypertrophic responses[32]. Additionally, many in vitro studies support the statement that integrin signaling takes part in cardiac hypertrophy[33]. Another factor related to integrins is integrin-linked kinase (ILK), which is a multifunctional protein kinase. ILK-regulated signaling is an adaptive hypertrophic response mechanism related to multiple clinical heart diseases[34].

Integrins are activated by ILK and bind to ligands that can induce intracellular signaling cascades that affect many cellular biological processes[35]. Specifically, integrins can transmit intracellular signals and play positive and negative roles in growth regulation by combining with many receptors in the ECM (extracellular matrix) including CAM (cell adhesion molecule) and LAMA4 (laminin α4 chain)[36]. The ECM is contact with force-generating proteins and the myocyte cytoskeleton through transmembrane proteins including integrins[37]. Previous studies have shown that interactions between integrins and their surrounding ECM are related to heart development and maturation[38]. In our present study, ITGA4 and ITGB8 were elevated after kisspeptin-10 treatment at the transcriptional and protein levels. Based on the contraction of cardiomyocytes through histological observations, it could be preliminarily speculated that kisspeptin-10 promotes myocardial contraction by changing integrin-ECM signaling.

Furthermore, a key downstream ECM kinase, focal adhesion kinase (FAK), is nvolved in the integrin-ECM signaling pathway[39]. To date, there is more evidence showing that integrin-FAK pathway plays a crucial role in cardiomyocytes under challenging conditions, such as transverse aortic constriction (TAC), angiotensin II stimulation and aging. The MYL7 gene is also involved in regulating the actin cytoskeleton pathway, which is related to heart diseases. Myosin regulation light chain 2 is encoded by the MYL7 gene and is also called atrial light chain-2 (ALC-2)[40]. ALC-2 is restricted to atria cardiac muscles in healthy individuals, where it modulates contractility and cardiac development. ALC-2 also regulates contractility and stabilizes thick filaments in the vertebrate heart[41]. Moreover, ALC-2 generally binds to alpha myosin heavy chain[42]. This protein is encoded by the MYH6 gene and is associated with atrial septal defects, late-onset hypertrophic cardiomyopathy and sick sinus syndrome due to mutations. Based on these results, it has been suggested that FAK is not only a protective factor against heart hypertrophy[43], but that it regulates the actin cytoskeleton pathway and is also required for normal heart function. In this study, elevated expression of the key gene MYL7 indicated that kisspeptin-10 treatment might activate the pathways related to MYL7 in rat heart.

To understand the multiple effects of kisspeptin-10 on the rat heart in depth, a metabolic approach was employed in this study. The disturbances in serum metabolites represent a metabolic view of systemic status dysfunction. Recently, plasma metabolite profiling using nuclear magnetic resonance spectroscopy, mass spectroscopy and GC-MS/TOF has facilitated new studies in cardiovascular diseases. In the present study, a GC-MS/TOF approach was applied to explore cardiac dysfunction and the post-transcriptional control and then to identify cardiovascular biomarkers and metabolic pathways in rats treated with kisspeptin-10. PCA Analysis (Fig 4) indicated that metabolites in the kisspeptin-10 group deviated from the control group, suggesting that significant systemic metabolite changes were induced by kisspeptin-10. Identification of differentially expressed metabolites and the related pathways that were induced by kisspeptin-10 was accomplished with a supervised pattern recognition called OPLS-DA. The results showed that the 26 differentially expressed endogenous metabolites were enriched in several pathways including carbohydrate metabolism, lipid metabolism, branched chain amino acid (BCAA) metabolism, glycogenic amino acid (GAA) metabolism, ornithine metabolism, tyrosine metabolism, methionine metabolism, cysteine metabolism and arachidonic acid metabolism. These pathways were classified into energy metabolism, amino acid metabolism, organic acid metabolism and other metabolisms and were selected from the KEGG pathway for further study. The perturbations of these pathways induced by kisspeptin-10 were involved in proteolysis, disruption of myocardium structure, cardiac hypertrophy, and inflammation.

### Energy metabolism

The heart must continuously generate a large amount of adenosine triphosphate (ATP) for energy to sustain its basal metabolic processes, systolic functions and ionic homeostasis. It is known that 60–90% of energy production for ATP synthesis is derived from fatty acid oxidation in the healthy heart. Previous studies have shown that the development of heart failure is associated with a shift from the dominant bioenergetic state of fatty acid oxidation to the more glycolytic state[20]. This is viewed as a compensatory response for promoting fuel utilization efficiency[44]. In our present study, the down regulation of galactose and up regulation of glycogenic amino acids (GAAs), including L-alanine, L-glutamic acid, L-methionine, L-proline and L-valine, in the kisspeptin-10 group suggests the accelerated carbohydrate metabolism, which indicates a negative energy balance. Moreover, in the kisspeptin-10-treated rats, high levels of GAAs that were possibly derived from protein decomposition might cause a negative nitrogen balance[45]. It was found that increases of ketogenic amino acids such as tyrosine indicate a disturbance in glucose use, which might be induced by kisspeptin-10 in this study. In contrast, cholesterol increased significantly in the rats treated with kisspeptin-10, suggesting disrupted lipid metabolism, which might result from a lipolysis blockade. According to a previous report, decreased fatty acid oxidation might be induced by damages to the mitochondrial electron transport chain. Meanwhile, histopathological evidence from our study showed mitochondrial damage via a transmission electron microscope (TEM). The likely hypothesis is that the metabolic switch and mitochondrial damage were results of kisspeptin-10 treatment.

### Amino acid metabolism

In the present study, a wide range of amino acids was identified as being differentially expressed between the kisspeptin-10 and control groups. To date, researchers have demonstrated that cardiomyopathy risks could be predicted by plasma amino acid profiling. Furthermore, Shah et al.[46] reported that branched chain amino acids (BCAAs) were involved in some cardiovascular events. These branched chain amino acids (BCAAs), including valine, leucine and isoleucine, are mostly catabolized in cardiac muscle, the kidneys and neurons[47]. Studies have shown that BCAAs are important sources for the biosynthesis of ketone bodies, sterols and glucose[48]. It is also known that BCAAs can inhibit the transportation and utilization of fatty acids and pyruvate[49], which is consistent with the elevated BCAAs and accumulation of pyruvate after treatment with kisspeptin-10 in our study. BCAAs are not merely an important nutrient source; they are also effective signaling molecules. For example, L-leucine is a highly effective activator of the mTOR signaling pathway[50]. In the heart, increased BCAA levels can activate mTOR signaling, which is directly implicated in cardiac hypertrophy through protein synthesis regulation[51, 52]. Moreover, mTOR activation induced by BCAAs suppresses autophagy, a known factor in cardiac pathologies[53, 54]. A previous study had demonstrated that the BCAAs can protect cardiac tissue against MI injury delay ischemic contracture in the depleted ischemic heart[49].

Meanwhile, the accumulation of glycogenic amino acids (GAAs) in the kisspeptin-10 group, including L-alanine, L-glutamic acid, L-methionine, L-proline and L-valine, could cause a negative nitrogen balance in the body state, which is likely derived from protein decomposition[45]. The present study also showed that urea levels were increased in the kisspeptin-10 group compared with the control group. According to previous reports, increased GAAs and urea suggests accelerated of protein disintegration for energy compensation[24].

Ornithine, which is derived from arginine, could be an upstream precursor for the formation of polyamines including spermine and spermidine. Our results demonstrated increased ornithine and spermidine, which is consistent with other studies that have shown that increased ornithine levels in heart failure (HF) patients might be involved in impaired NO synthesis and promoting polyamine synthesis. Polyamines are also increased in many experimentally induced cardiac hypertrophy models[55]. Specifically, spermidine has been reported to be unfavorable for cardiomyopathy during hypoxia stress[56]. Additionaly, the NO and polyamine pathway are inter-regulated. NO can inhibit polyamine production by controlling ornithine decarboxylase[57]. NO also stimulates guanylate cyclase, which acted as an intracellular messenger, and consequently relaxes smooth muscle in blood vessels[17]. Ornithine metabolism and the histopathological HE staining result from our study suggest that the irregular cardiac muscle contraction and stretch functions might be a result of impaired NO synthesis in the ornithine metabolism pathway.

Our study detected that serum tyrosine level was increased in the kisspeptin-10 group. Tyrosine accumulation is indicative of tetrahydrobiopterin depletion during the progression of heart failure. Tetrahydrobiopterin depletion in the myocardium contributes to heart failure in human subjects and animal models[58].

High methionine level was also detected in the kisspeptin-10 group in our study. Studies have shown that methionine acts as an endogenous antioxidant by relieving ROS-induced injury[59]. Therefore, the elevated methionine might play a protective role after kisspeptin-10 treatment. g

The increased level of hypotaurine is derived from the reduction of excess cysteine that can synthesis GSH. And then, GSH can combine with oxygen radicals (ROS) to form GSSH against oxidative stress. This indicates that kisspeptin-10 might induce cysteine metabolism to protect against cardiac oxidative stress in our study.

### Other metabolism

Arachidonic acid level was elevated after kisspeptin-10 treatment in our study, which is consistent with a previous report that showed that kisspeptin could release arachidonic acid[8, 60]. Moreover, arachidonic acid accumulation has been shown to increase the risk of non-fatal acute myocardial infarction (MI) and is regarded as an important biomarker for the onset of MI[61]. Pathological disorders induced by increased arachidonic acid are involved in inflammation and disrupting the cell membrane[16].

## Conclusion

**Fig 8:**
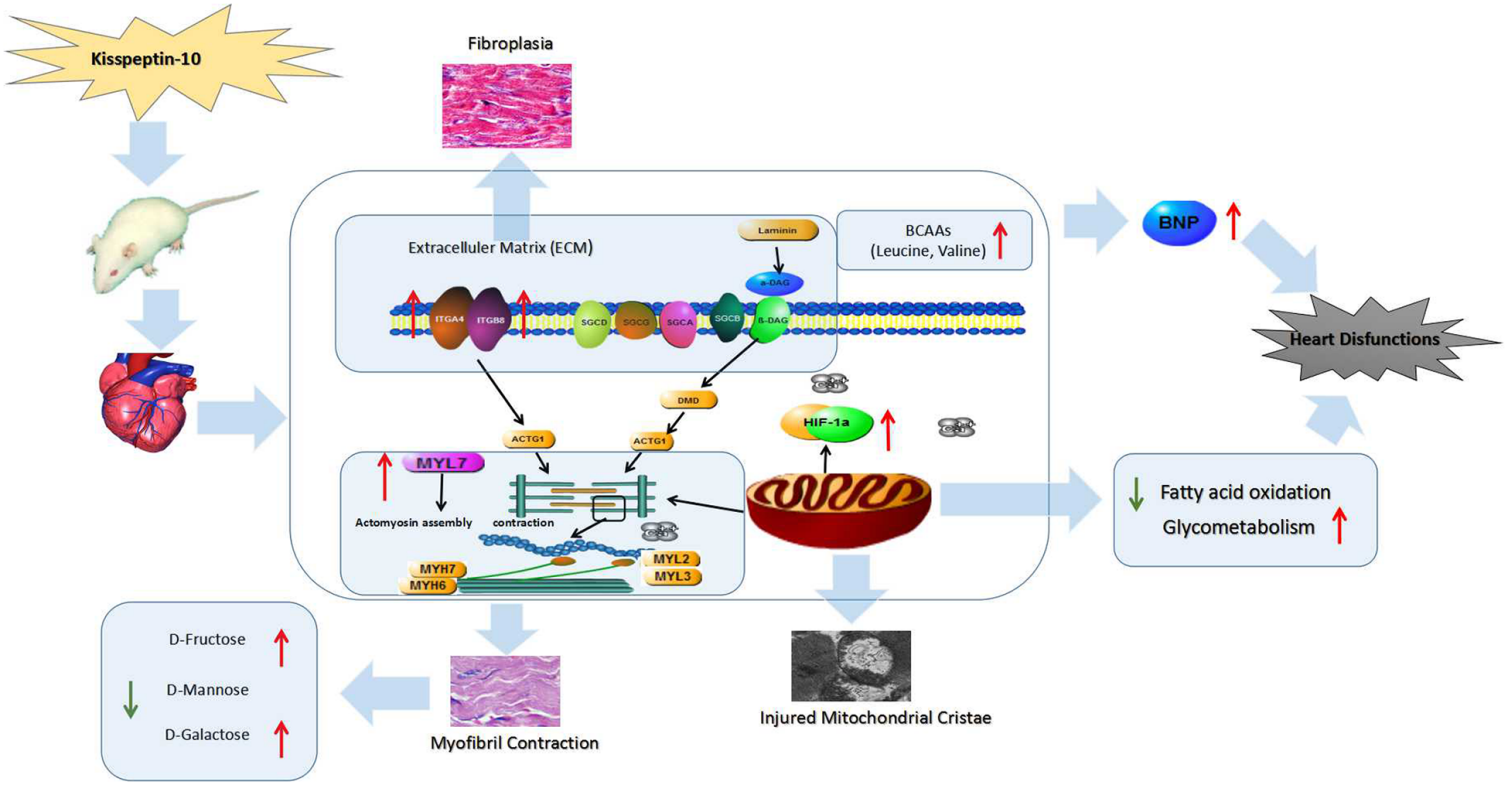
Changes in serum metabolite concentration, heart tissue morphology, and mRNA and protein expression levels of key genes were induced by kisspeptin-10 compared with the control group. All of the factors revealed that kisspeptin-10 induced myocardial dysfunctions.

In the present work, the multiple effects of kisspeptin-10 on heart function were evaluated from four aspects, including the histopathological, gene transcriptional, protein expression and metabolic levels in rats. These four aspects were combined to better understand the roles and mechanistic effects of kisspeptin-10 on heart function. The histopathological observations demonstrated myocardial changes and cardiomyocyte damage via HE and Masson staining and transmission electron microscope. The appearance of histopathological features, including wavy myocardial contractions, mitochondrial cristae fracture and a certain degree of myocardial fibrosis, were in line with variation in several genes and proteins involved in heart diseases pathways. The alterations in key genes, proteins and morphological features resulting from kisspeptin-10 treatment correlated with the observed metabolite variation. The 26 significantly altered metabolites between the kisspeptin-10 and control groups could be regarded as potential biomarkers of heart diseases induced by kisspeptin-10. In summary, the present study provided novel insight into the influence of kisspeptin-10 on heart functions and it can be summarized in Fig 8. Our work also expands the current knowledge on kisspeptin-10-based effects on the heart, which could further lead to the development of therapeutics related to kisspeptin-10 including antagonists and analogs.
